# Supplementary material for: Resolving the full spectrum of human genome variation using Linked-Reads
Source: Genome Res. 2019 Apr;29(4):635–45. doi: 10.1101/gr.234443.118 (PMC6442396; doi:10.1101/gr.234443.118)
Supplement: Supplemental Material [file supp_gr.234443.118_Supplemental_longranger-2.2.zip › longranger-2.2.tar.bz2!/lr2.2-public/lib/stan/cmdstan/stan/doc/api/html/stepsize__var__adapter_8hpp_source.html]

Stan: src/stan/mcmc/stepsize\_var\_adapter.hpp Source File


|  |  |
| --- | --- |
|  | Stan  2.5.0  probability, sampling & optimization |


- Main Page
- Related Pages
- Namespaces
- Classes
- Files

- File List
- File Members

All Classes Namespaces Files Functions Variables Typedefs Enumerations Enumerator Friends Macros Pages

- src
- stan
- mcmc

stepsize\_var\_adapter.hpp

Go to the documentation of this file.

1 #ifndef STAN\_\_MCMC\_\_STEPSIZE\_\_VAR\_\_ADAPTER\_\_BETA

2 #define STAN\_\_MCMC\_\_STEPSIZE\_\_VAR\_\_ADAPTER\_\_BETA

3

4 #include <stan/mcmc/base\_adapter.hpp>

5 #include <stan/mcmc/stepsize\_adaptation.hpp>

6 #include <stan/mcmc/var\_adaptation.hpp>

7

8 namespace stan {

9

10  namespace mcmc {

11

12  class stepsize\_var\_adapter: public base\_adapter {

13

14  public:

15

16  stepsize\_var\_adapter(int n): var\_adaptation\_(n)

17  {};

18

19  stepsize\_adaptation& get\_stepsize\_adaptation() {

20  return stepsize\_adaptation\_;

21  }

22

23  var\_adaptation& get\_var\_adaptation() {

24  return var\_adaptation\_;

25  }

26

27  void set\_window\_params(unsigned int num\_warmup,

28  unsigned int init\_buffer,

29  unsigned int term\_buffer,

30  unsigned int base\_window,

31  std::ostream\* e = 0) {

32  var\_adaptation\_.set\_window\_params(num\_warmup,

33  init\_buffer,

34  term\_buffer,

35  base\_window,

36  e);

37  }

38

39

40  protected:

41

42  stepsize\_adaptation stepsize\_adaptation\_;

43  var\_adaptation var\_adaptation\_;

44

45  };

46

47  } // mcmc

48

49 } // stan

50

51 #endif

stan::mcmc::stepsize\_adaptation

**Definition:** stepsize\_adaptation.hpp:11

stan::mcmc::var\_adaptation

**Definition:** var\_adaptation.hpp:14

stan::mcmc::base\_adapter

**Definition:** base\_adapter.hpp:8

stan::mcmc::stepsize\_var\_adapter

**Definition:** stepsize\_var\_adapter.hpp:12

stan::mcmc::stepsize\_var\_adapter::stepsize\_adaptation\_

stepsize\_adaptation stepsize\_adaptation\_

**Definition:** stepsize\_var\_adapter.hpp:42

stan::mcmc::stepsize\_var\_adapter::get\_var\_adaptation

var\_adaptation & get\_var\_adaptation()

**Definition:** stepsize\_var\_adapter.hpp:23

stan::mcmc::windowed\_adaptation::set\_window\_params

void set\_window\_params(unsigned int num\_warmup, unsigned int init\_buffer, unsigned int term\_buffer, unsigned int base\_window, std::ostream \*e=0)

**Definition:** windowed\_adaptation.hpp:32

stan::mcmc::stepsize\_var\_adapter::stepsize\_var\_adapter

stepsize\_var\_adapter(int n)

**Definition:** stepsize\_var\_adapter.hpp:16

stan::mcmc::stepsize\_var\_adapter::get\_stepsize\_adaptation

stepsize\_adaptation & get\_stepsize\_adaptation()

**Definition:** stepsize\_var\_adapter.hpp:19

stan::mcmc::stepsize\_var\_adapter::set\_window\_params

void set\_window\_params(unsigned int num\_warmup, unsigned int init\_buffer, unsigned int term\_buffer, unsigned int base\_window, std::ostream \*e=0)

**Definition:** stepsize\_var\_adapter.hpp:27

stepsize\_adaptation.hpp

stan::math::e

double e()

Return the base of the natural logarithm.

**Definition:** constants.hpp:86

var\_adaptation.hpp

stan::mcmc::stepsize\_var\_adapter::var\_adaptation\_

var\_adaptation var\_adaptation\_

**Definition:** stepsize\_var\_adapter.hpp:43

base\_adapter.hpp


---

[ Stan Home Page ]

*© 2011–2014,
Stan Development Team.*
